# First Robotic Reconstructive Urology Experience in Indonesia: Early Outcomes from a National Center

**DOI:** 10.5152/tud.2026.26062

**Published:** 2026-10-13

**Authors:** Adhitama Alam Soeroto, Muhammad Andi Iqbal Maulana, Stevan Kristian Lionardi, Irfan Wahyudi

**Affiliations:** Department of Urology, Cipto Mangunkusumo National General Hospital, Universitas Indonesia Faculty of Medicine, Jakarta, Indonesia

**Keywords:** Reconstructive surgical procedures, robotic surgical procedures, trauma and reconstructive surgery, ureter, urinary fistula

## Abstract

**Objective:**

Robotic reconstructive urology is well described on the da Vinci system but rarely on other platforms, and seldom from low- and middle-income countries. We report an initial experience with the Edge MP1000 and Toumai multiport systems at a national referral center.

**Methods:**

We retrospectively reviewed 7 consecutive adults who underwent robot-assisted reconstruction for benign ureteral or fistulous disease between October 28, 2024, and July 31, 2025. Outcomes were docking and operative times, conversion, estimated blood loss (EBL), length of stay (LOS), 30-day Clavien–Dindo complications, and early functional success, defined as freedom from ureteral stents and nephrostomy tubes with imaging-confirmed patency.

**Results:**

All 7 procedures were completed robotically. The cohort comprised 3 benign ureteral strictures, 1 combined distal stenosis reconstructed with a Boari flap, 2 ureterovaginal fistulas, and 1 vesicouterine fistula. The median age was 39 years, docking time 10 minutes, operative time 250 minutes, EBL 50 mL, and LOS 6 days. One Clavien–Dindo grade II complication occurred, with no higher grade, no reoperation, and no death. Every patient achieved early functional success at a median follow-up of 5 months, hydronephrosis resolved in all 5 affected kidneys, and renal function was stable in 6 patients. Docking and operative times fell in the later cases, but platform and case complexity changed together.

**Conclusion:**

In this small single-center series, benign urinary tract reconstruction was completed safely on the Edge MP1000 and Toumai systems. These findings are preliminary and should not be generalized. Longer follow-up is needed to establish durability.

**Main Points**

- Seven consecutive benign urinary tract reconstructions were completed on the Edge MP1000 and Toumai systems without conversion to open or laparoscopic surgery.
- One Clavien–Dindo grade II complication occurred, and every repair was patent and free of ureteral stents and nephrostomy tubes at the most recent follow-up, a median of 5 months.
- A Boari flap and a buccal mucosal graft ureteroplasty were completed on systems whose published urologic record is almost entirely oncologic.
- Docking and operative times fell across the series, but platform, case complexity, and team experience changed together, so no platform effect can be claimed.

## Introduction

Robot-assisted surgery is established in urology, particularly for procedures that benefit from three-dimensional vision, wristed instrumentation, and surgeon ergonomics. In ureteral reconstruction, systematic reviews report high patency with low morbidity, supporting robotic repair as an alternative to open or conventional laparoscopic surgery in selected patients.^1,2^ Benign ureteral strictures and genitourinary fistulas cause recurrent infection, pain, obstruction, and progressive loss of renal function, often in young patients, and durable repair depends on careful dissection and a tension-free anastomosis.^1^

Most published experience in robotic urology comes from high-income settings and from cancer surgery. In low- and middle-income countries (LMICs), adoption is limited by acquisition and maintenance costs, restricted reimbursement, and the absence of sustainable financing models. Uptake also tracks market pressure, and Wright et al found that hospital competition predicted adoption of robot-assisted surgery.^3^ Robotic surgery is therefore available in few centers, often at substantial out-of-pocket cost to patients.^4^

Starting a program in an LMIC depends on infrastructure, trained staff, and dependable technical support, none of which can be assumed even in large urban centers. Proficiency builds over successive cases,^5^ so structured training pathways combining simulation, graded responsibility, and supervised practice are required when a program begins.^6^

Urologic reports of the Toumai and Edge MP1000 systems cover radical prostatectomy and partial nephrectomy and describe acceptable perioperative outcomes.^7,8^ Their performance in complex benign reconstruction has not been reported.

Our institution manages a high volume of complex reconstructive urology cases and recently introduced both systems. We describe an initial experience with robot-assisted ureteral reconstruction and fistula repair at a national referral center in an LMIC, with a focus on perioperative safety and early functional recovery.

## Material and Methods

This retrospective, single-center observational study was conducted in the department of urology of a national referral center between October 28, 2024, and July 31, 2025. Over this period, 7 consecutive adults underwent robot-assisted reconstruction. Two reconstructive urologists, both with prior open and laparoscopic experience, performed all operations. Data were drawn from a prospectively maintained institutional database and de-identified before analysis.

This study was approved by the Health Research Ethics Committee of the Faculty of Medicine, Universitas Indonesia – Dr. Cipto Mangunkusumo National General Hospital (approval number 25-08-1196, dated October 6, 2025), and followed the principles of the Declaration of Helsinki as revised in 2024. All participants gave written informed consent for surgery and for the use of de-identified clinical data in research and publication.

This study used Claude (version Opus 5.0; Anthropic, San Francisco, CA, USA) for grammatical improvement and English language editing during the preparation of the manuscript. However, all content of the study has been reviewed and edited by the authors following the use of AI/LLM tools.

### Patient Selection and Indications

Patients were eligible if they were aged 18 years or older, had benign ureteral pathology or a genitourinary fistula suitable for minimally invasive repair, and were fit for general anesthesia and able to tolerate pneumoperitoneum. Every consecutive eligible patient treated in the study period was included, and none declined.

### Data Collection and Preoperative Assessment

Baseline variables were age, sex, body mass index (BMI), diagnosis, etiology, stricture level and length, and presenting clinical features.

Every patient underwent a standard preoperative workup comprising history, examination, and routine laboratory tests (complete blood count, serum creatinine, electrolytes, and coagulation profile). Cross-sectional imaging (computed tomography [CT] urography or magnetic resonance imaging [MRI]) with renal ultrasound defined the level and length of ureteral disease, the degree of hydronephrosis, and renal morphology. Baseline renal function was assessed with serum creatinine and estimated glomerular filtration rate (eGFR). All 3 patients with fistulous disease underwent contrast CT urography and cystoscopy before surgery, together with a methylene blue instillation test to exclude a vesicovaginal fistula. Postoperative assessment comprised clinical review and renal ultrasound, and neither CT urography nor a repeat methylene blue test was performed after surgery.

### Robotic Platforms and Surgeon Experience

Operations used the Edge MP1000 platform (Edge Medical Robotics Co., Ltd., Shenzhen, China) in phase 1 and the Toumai platform (Shanghai MicroPort MedBot Co., Ltd., Shanghai, China) in phase 2. Allocation followed availability and scheduling rather than case selection. A da Vinci system was not available at our institution during the study period, so no contemporaneous robotic comparator existed. Both surgeons had completed formal robotic training before the program began. Each case was led by a console surgeon supported by a bedside assistant urologist, and this pairing was unchanged across the series. Operative steps were standardized between the 2 surgeons and followed techniques described in published robotic series.^1,2^ Stricture length was measured on preoperative cross-sectional imaging and confirmed at dissection.

### Perioperative Management and Follow-Up

Perioperative care followed institutional enhanced recovery pathways. Patients received prophylactic antibiotics and venous thromboembolism prophylaxis according to local protocols, were mobilized from postoperative day 1, and resumed diet as tolerated.

At the end of each procedure, a urethral catheter was placed, together with a double-J ureteral stent or nephrostomy tube when appropriate. Drains were used selectively and removed once output was low and serous. Length of stay (LOS) was calculated from the day of surgery to discharge.

Patients were reviewed at 1 to 3 months, at approximately 3-monthly intervals thereafter, and as needed, with clinical assessment, serum creatinine and eGFR, and renal ultrasound. Antegrade or retrograde studies and CT urography were added when required to confirm ureteral patency and improvement of hydronephrosis. Patency was defined as unobstructed contrast passage on an antegrade or retrograde study, or, where no contrast study was performed, as the absence of obstructive hydronephrosis on ultrasound or cross-sectional imaging. Hydronephrosis was graded on ultrasound using the Society for Fetal Urology classification, and its change from baseline was recorded as resolved, improved, unchanged, or worsened. No patient was lost to follow-up.

### Outcome Measures

Recorded variables were operative time (skin incision to closure), docking time (final cart positioning to completion of all robotic arm attachments), estimated blood loss (EBL), LOS, and perioperative complications. Feasibility was judged by docking time, total operative time, and any conversion to open surgery or conventional laparoscopy. Safety was assessed by EBL, LOS, and 30-day complications graded with the Clavien–Dindo classification, with grade II or higher regarded as clinically significant.

Early functional success was defined as freedom from ureteral stents and nephrostomy tubes at the most recent follow-up, with imaging-confirmed patency and no need for further intervention. Change in hydronephrosis and in eGFR relative to baseline was also recorded.

Cases were grouped by phase, phase 1 covering the Edge MP1000 cases of October to November 2024 and phase 2 the Toumai cases of June to July 2025. The grouping follows the order in which the 2 systems were introduced and was prespecified as descriptive.

### Statistical Analysis

Only descriptive statistics were used. Continuous variables are presented as median (interquartile range [IQR]), computed with the default quantile method (type 7, linear interpolation), and categorical variables are presented as counts and percentages. Data were compiled and analyzed in Microsoft Excel. Reporting followed the STROBE statement for observational studies.^9^

## Results

### Patient Characteristics and Indications

Seven consecutive patients were treated during the study period. The median age was 39 years (IQR: 37-45), and the median BMI was 26.2 kg/m^2^ (IQR: 25.6-28.0). One patient had a BMI of 36.4 kg/m^2^, and the procedure was completed robotically. The cohort included 6 women and 1 man.

Indications were 3 benign ureteral strictures, 1 combined distal stenosis reconstructed with a Boari flap, 2 ureterovaginal fistulas, and 1 vesicouterine fistula. Three ureteral lesions were proximal and 3 distal, and where a stricture length applied, the median was 2.0 cm (IQR: 2.0-4.25; range: 2.0-8.0). Most injuries were iatrogenic, following gynecologic surgery in 4 patients and endourologic procedures in 2, with 1 of uncertain cause. Procedures were ureteroneocystostomy with or without a Boari flap in 4 patients, ureteroureterostomy in 1, buccal mucosal graft ureteroplasty in 1, and vesicouterine fistula repair in 1 (Table 1).

**Table 1.** Patient, Perioperative, and Short-Term Follow-Up Characteristics (n = 7)

Case	Phase	Platform	Procedure	Age (y)	BMI (kg/m^2^)	Stricture Length (cm)	Operative Time (min)	Docking Time (min)	EBL (mL)	LOS (d)	FU (mo)	Clavien–Dindo (≤30 d)	Hydronephrosis (pre to post)	Stent- and Nephrostomy-Free, Patent
1	Phase 1 (Oct 2024)	Edge MP1000	Ureteroureterostomy (R)	46	36.4	2	250	20	150	7	12	0	Grade 1 to resolved	Yes
2	Phase 1 (Oct 2024)	Edge MP1000	Ureteroneocystostomy (R) for ureterovaginal fistula	35	25.6	2	290	14	30	5	2	0	None to grade 1 right	Yes
3	Phase 1 (Nov 2024)	Edge MP1000	Vesicouterine fistula repair	32	28.3	NA	350	10	70	5	1	0	None to none	Yes
4	Phase 1 (Nov 2024)	Edge MP1000	Ureteroneocystostomy (L) for ureterovaginal fistula	52	25.7	2	230	10	50	5	21	2	Grade 1 to resolved	Yes
5	Phase 2 (Jun 2025)	Toumai	Buccal mucosal graft ureteroplasty (R)	39	27.7	5	390	6	50	7	12	0	Grade 2 to resolved	Yes
6	Phase 2 (Jul 2025)	Toumai	Ureteroneocystostomy (L)	39	20.8	2	190	5	100	7	4	0	Grade 3 to resolved	Yes
7	Phase 2 (Jul 2025)	Toumai	Ureteroneocystostomy plus Boari flap (R)	44	26.2	8	220	6	50	6	5	0	Grade 2 to resolved	Yes
Phase 1 (n = 4)				40.5 (34.3-47.5)	27.0 (25.7-30.3)	2 (2-2)	270 (245-305)	12 (10-15.5)	60 (45-90)	5 (5-5.5)	7 (1.75-14.25)	1	—	4/4
Phase 2 (n = 3)				39 (39-41.5)	26.2 (23.5-26.9)	5 (3.5-6.5)	220 (205-305)	6 (5.5-6)	50 (50-75)	7 (6.5-7)	5 (4.5-8.5)	0	—	3/3

BMI, body mass index; EBL, estimated blood loss; FU, follow-up; IQR, interquartile range; L, left; LOS, length of stay; NA, not applicable (fistula repair, no stricture length); R, right. Phase 1 comprised the Edge MP1000 cases, and phase 2 the Toumai cases. Summary rows report median (IQR), computed with the default quantile method (type 7, linear interpolation). The Clavien–Dindo column gives the highest grade complication within 30 d (0 = none). Hydronephrosis compares preoperative and latest postoperative ultrasound.

### Perioperative Outcomes

No procedure required conversion to open surgery or conventional laparoscopy. The Edge MP1000 platform was used in the 4 phase 1 cases, and the Toumai platform was used in the 3 phase 2 cases. Docking took a median of 10 minutes (IQR: 6-12), and operations a median of 250 minutes (IQR: 225-320). The median EBL was 50 mL (IQR: 50-85), and the median postoperative LOS 6 days (IQR: 5-7).

In phase 1, the median docking time was 12 minutes (IQR: 10-15.5) and the median operative time 270 minutes (IQR: 245-305). In phase 2, these were 6 minutes (IQR: 5.5-6.0) and 220 minutes (IQR: 205-305), respectively. The median EBL was 60 mL (IQR: 45-90) in phase 1 and 50 mL (IQR: 50-75) in phase 2, and the median LOS was 5 days (IQR: 5-5.5) and 7 days (IQR: 6.5-7) in phases 1 and 2, respectively. The longer stay in phase 2 can be attributed to the more complex reconstructions performed then, the Boari flap, and the buccal mucosal graft ureteroplasty (Table 1).

### Complications

Within 30 days, 1 patient had a Clavien–Dindo grade II complication managed with medication alone. There were no grade III-V complications, reoperations, or readmissions, and there were no perioperative deaths.

### Functional Outcomes and Follow-Up

The median follow-up was 5 months (IQR: 3-12, range: 1-21). Every patient met the definition of early functional success, with patency confirmed on cross-sectional imaging or renal ultrasound. Hydronephrosis resolved in all 5 kidneys affected before surgery, which ranged from Society for Fetal Urology (SFU) grade 1 to grade 3. One patient without preoperative hydronephrosis developed SFU grade 1 right hydronephrosis on ultrasound 12 days after ureteral stent removal, which was asymptomatic and required no intervention. Serum creatinine and eGFR were stable in 6 patients. In 1 patient with pre-existing chronic kidney disease after bilateral nephrostomy, percutaneous nephrolithotomy, and lasertripsy, eGFR fell from 47.5 to 38.5 mL/min/1.73 m^2^ and creatinine rose from 1.83 to 2.1 mg/dL at 3 months, with no hydronephrosis on ultrasound to suggest obstruction.

All patients reported improvement or resolution of their presenting symptoms. The vesicouterine fistula occurred in a woman with a 4-year history of cyclical hematuria and urinary leakage after 2 cesarean deliveries, and both symptoms resolved without recurrence. In the 2 ureterovaginal fistulas, the continuous urinary leakage stopped after ureteroneocystostomy. The buccal mucosal graft ureteroplasty was performed for a 5-cm proximal stricture after previous endourologic treatment, demonstrated on preoperative retrograde pyelography (Figure 1). A 55 × 15 mm graft from the left cheek was used as a ventral onlay reinforced with an omental wrap (Figure 2). Renal ultrasound at 12 months showed no hydronephrosis (Figure 3).

**Figure 1.**
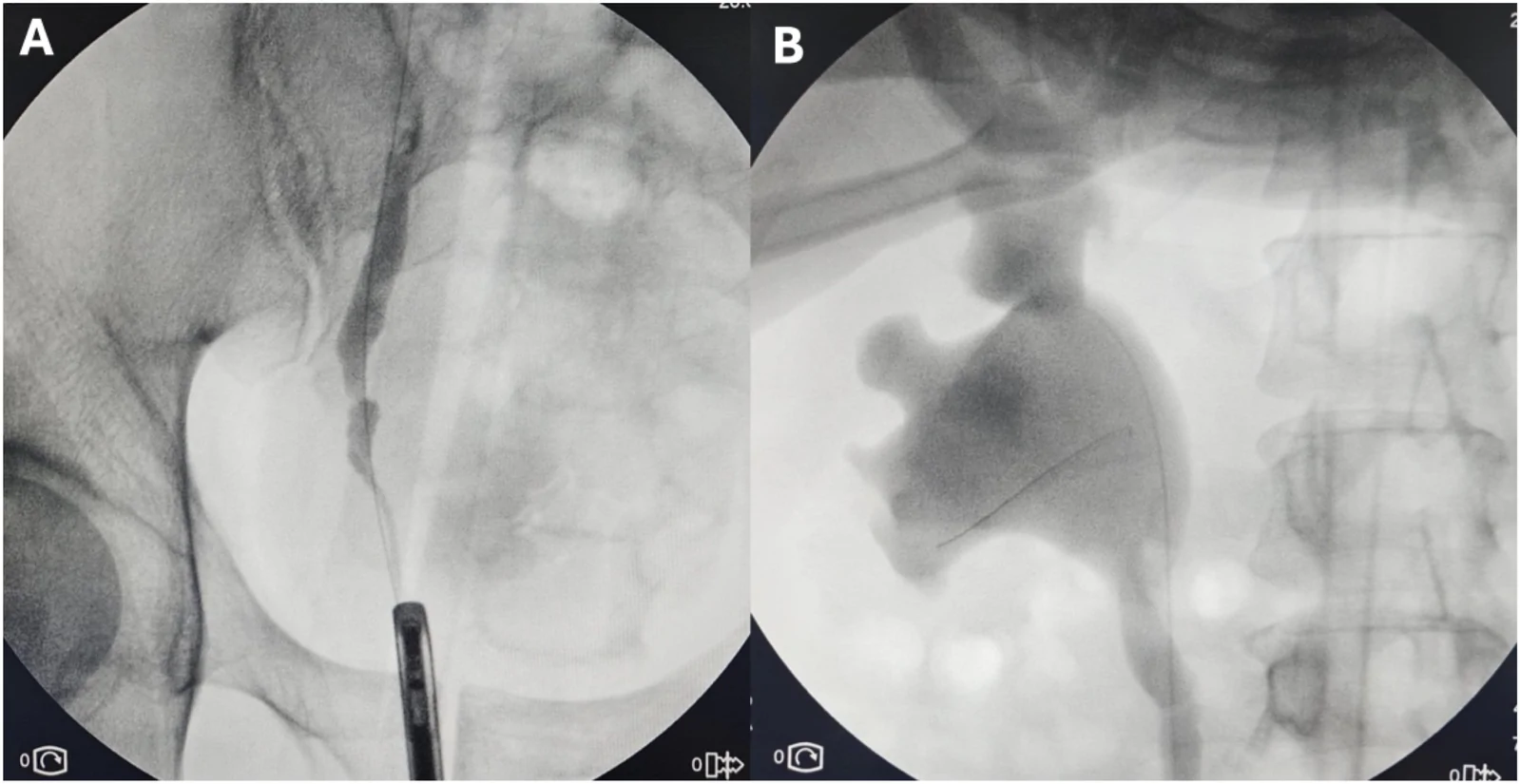
Preoperative retrograde pyelography. (A) Stricture with ureteric catheter *in situ*. (B) Pelvicalyceal system proximal to the stricture.

**Figure 2.**
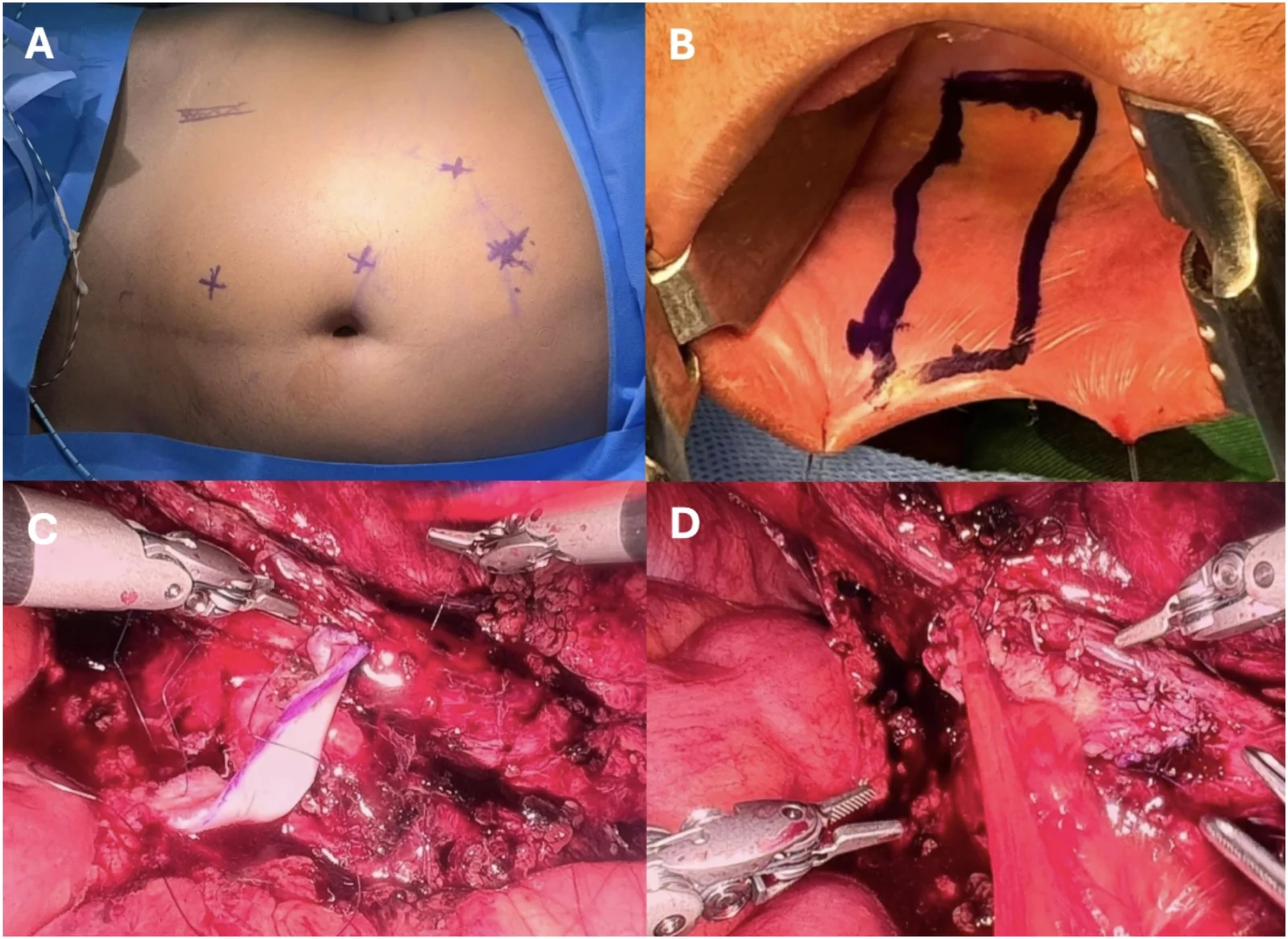
Robot-assisted buccal mucosal graft ureteroplasty. (A) Port sites. (B) Buccal graft outlined before harvest. (C) Graft alongside the spatulated ureter. (D) Completed ventral onlay.

**Figure 3.**
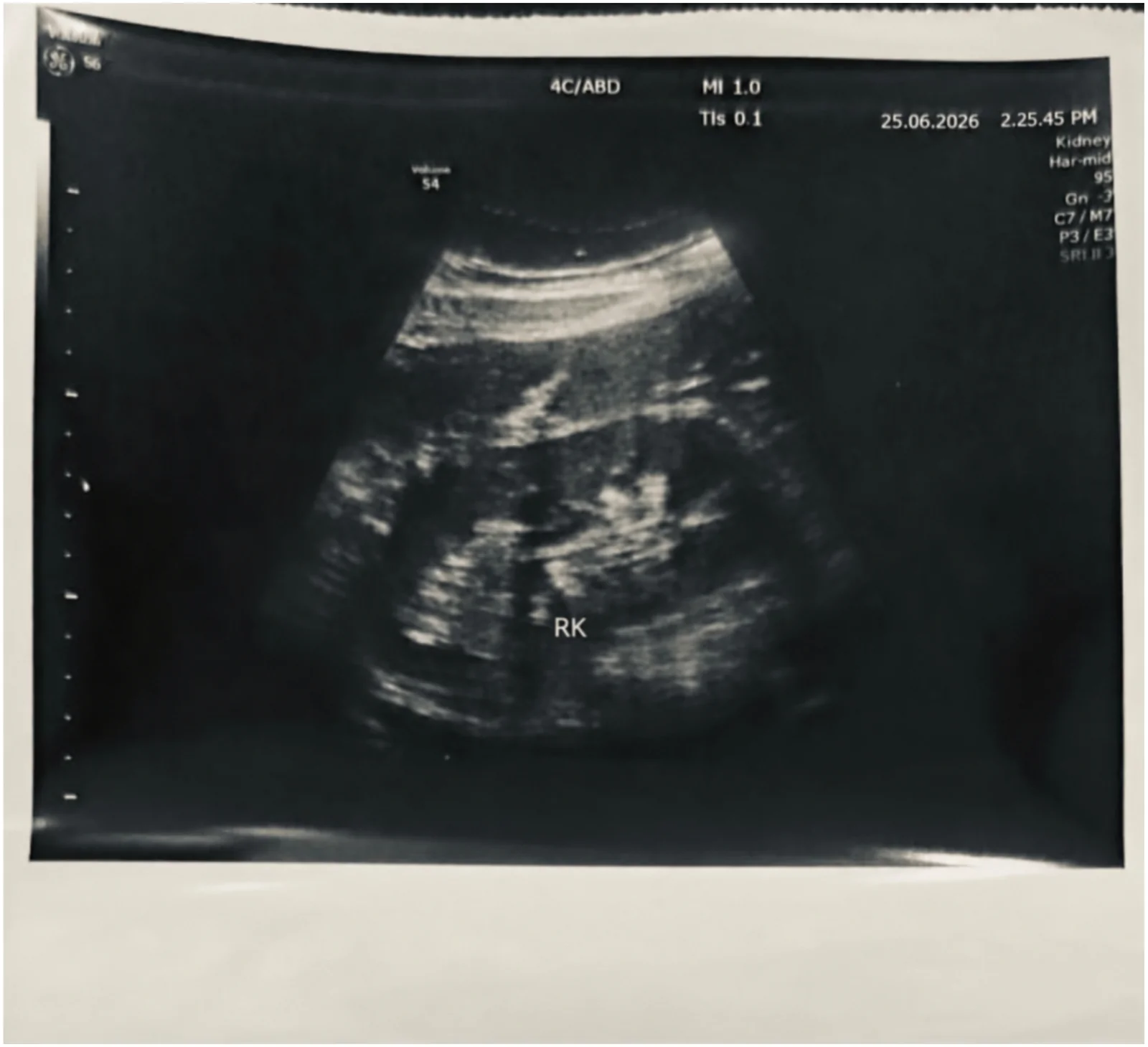
Renal ultrasound at 12 months showing no hydronephrosis. RK, right kidney.

## Discussion

Seven consecutive benign reconstructions were completed on 2 multiport systems whose published urologic record is almost entirely oncologic,^7,8^ and we are not aware of any prior report of either system used for complex benign reconstruction of the upper or lower urinary tract. No case required conversion, which we attribute to cautious selection at the start of the program as much as to the systems themselves.

Reported radiographic patency after robotic ureteral reconstruction ranges from 85% to 95% at 1-2 years, although success is defined in different ways.^10,11^ Recurrence after robotic upper tract repair is driven mainly by prior radiotherapy.^12^ Tyagi et al reported radiographic success in 41 of 44 ureters after robot-assisted reimplantation,^13^ and Rossanese et al^14^ found no failures in the robotic arm of a cohort reconstructed for iatrogenic ureteral injury at mid-term follow-up. Because most injuries in this series were iatrogenic, longer follow-up is needed to establish whether patency persists, particularly after the buccal mucosal graft and Boari flap repairs.^11,15^

Three procedures extend what has been reported on these systems. Two ureterovaginal fistula repairs and 1 vesicouterine fistula repair were completed robotically with resolution of symptoms, and no existing report of these systems describes fistula surgery.^16,17^ Buccal mucosal graft ureteroplasty is among the more demanding upper tract reconstructions,^11^ and it was completed without complication. Four conditions mattered for centers planning a similar program. Surgeons were already experienced in open and laparoscopic reconstruction, the console and bedside team was stable, case selection was cautious at the start, and complex work was concentrated at a referral center so that caseload and referral pathways developed together.^5^ Sustained expansion will depend on reimbursement and financing, not on technique,^4,18^ and structured training with formal credentialing remains necessary as a program grows.^6,19^

This series is small, single center, and mixed in case type, which precludes conclusions about any single procedure or about either platform and limits generalizability to other centers and other case mixes. The median follow-up was 5 months, and although 3 patients have now been followed up for 12 months or longer, the remainder have not yet reached the 6- to 24-month window in which recurrences typically appear.^12,20^ Platform, surgeon familiarity, and case complexity changed together between the 2 phases, so the phase comparison is descriptive and permits no inference about the relative performance of the 2 systems. There was no open, laparoscopic, or da Vinci comparator. Larger multicenter studies with longer follow-up, agreed outcome definitions, and prospective cost analysis are needed to define the place of these systems in reconstructive urology in middle-income settings.^11^

Robotic reconstruction was introduced safely at an LMIC national referral center across a mixed caseload of benign ureteral and fistula disease, with low morbidity and functional success in every patient. Shorter operative times later in the series most likely reflect team familiarity, which this design cannot separate from platform or case mix. These findings describe what 7 cases required at a single center. They establish neither generalizability nor durability.

## Data Availability

The data that support the findings of this study are available on request from the corresponding author.
